# Complications rate of and risk factors for the unplanned reoperation of degenerative lumbar spondylolisthesis in elderly patients: a retrospective single-Centre cohort study of 33 patients

**DOI:** 10.1186/s12877-020-01717-2

**Published:** 2020-08-24

**Authors:** Weiyang Zhong, Xinjie Liang, Xiaoji Luo, Tianji Huang, Zhengxue Quan

**Affiliations:** 1 Department of Orthopedic Surgery, The First Affiliated Hospital of Chongqing, Medical University, Chongqing, China; 2 Department of Pain Management, The First Affiliated Hospital of Chongqing, Medical University, Chongqing, China

**Keywords:** Degenerative lumbar spondylolisthesis, Unplanned operation, Risk factor, Elderly patients

## Abstract

**Background:**

The study was to investigate the complications rate of and risk factors for unplanned reoperation among elderly patients who underwent posterior lumbar fusion (PLF) for degenerative lumbar spondylolisthesis (DLS).

**Methods:**

A total of 1100 DLS patients who were older than 60 years were reviewed from January 2006 to December 2016. 33 patients underwent unplanned reoperations and were analysed and divided into two groups (group A: posterolateral fusion, 650 patients; group B: intervertebral fusion, 450 patients). Sex, body mass index (BMI), radiographic data and clinical outcome data were analysed to evaluate the complications rate of and the risk factors for unplanned reoperations.

**Results:**

A total of 33 patients underwent unplanned reoperations (3%). The patients were followed up for an average of 4.20 ± 2.25 years (group A) and 4.32 ± 2.54 years (group B) without a significant difference. Significant differences were found in mean age, levels of involvement, hospital stay, surgery time, and blood loss between the groups. The causes of unplanned operation were wound infection, screw misplacement, neurological deficit, nonunion, and screw fracture, which were significant except for wound infection between the groups. Higher BMI (obesity), diabetes mellitus (DM), more bleeding and sex (female) were risk factors for complications. Cases of screw misplacement, neurological deficit, nonunion and screw fracture in group A were more significant than those in group B.

**Conclusion:**

Patients with higher BMI, DM, older age, posterolateral fusion, and female sex predicted a higher incidence of unplanned reoperations. Spine surgeons may need to pay more attention to their preoperative training and to improving surgical techniques that could reduce the reoperation rate.

## Background

With increases in the ageing population, the incidence of degenerative lumbar spondylolisthesis (DLS) and its surgical treatments has also received increasing attention because the rapid progression of anaesthesiology and surgical instruments has led to active surgical management in elderly patients [1]. However, preventing revision spinal surgery is still a matter of cardinal significance for spine surgeons and their patients. Unplanned reoperation is due to an unexpected event, the presence of persistent symptoms, the deterioration of previous potential diseases, or complications related to the primary operation [2, 3]. A better understanding of the complications rate of and risk factors for unplanned reoperations may help improve surgical outcomes and prognoses. It was reported that the cumulative reoperation rate in lumbar degenerative diseases was 4.7% at 3 months, 6.1% at 1 year, 8.5% at 2 years, 15.2% at 3 years, 17.7% at 5 years and 23.3% (38/163 patients) at the final follow-up [4–8]. Reducing the rate of lumbar surgery revision is also vital because the outcomes of DLS reoperation may be worse than the results of the initial surgical management.

Therefore, the study was to investigate the incidence of unplanned reoperation in elderly patients who underwent surgery for DLS and to analyse the complications rate and risk factors.

## Methods

### Patient selection

The study was approved by the institutional review board of our hospital. A total of 1100 patients were retrospectively reviewed, and the unexpected reasons for the unplanned reoperation were recorded and assessed. Between January 2006 and December 2016, 33 reoperation patients with DLS, aged more than 60 years old, were enrolled.

The inclusion criteria were one-level or two-level DLS requiring surgical treatment again. All the surgeries were performed for posterior lumbar fusion, including posterolateral fusion (650 patients) and intervertebral fusion (450 patients). Unplanned surgery was defined as reoperation in the operating room after the primary surgery. The primary and revision surgeries were all performed by the same senior surgical team. The exclusion criteria were as follows: patients who suffered from lumbar disc herniation, lumbar spinal stenosis, lumbar vertebral fractures, spondylitis or tumours.

Patient demographic data, including the primary surgical procedure and revision surgery information, were recorded in our study. The reasons for the unplanned surgery were assessed, and the reoperation rates of DLS were calculated.

### Statistical analysis

All statistical data were analysed with SPSS version 22.0 statistical software (SPSS, Inc., Chicago, IL, USA). The data were analysed by Student’s t-test for continuous variables and the Chi-square test for categorical variables. Values of *P* < 0.05 were regarded as statistically significant. For all regression models, a *p* value less than 0.05 with a confidence interval (CI) of 95% was considered significant.

## Results

**Fig. 1:**
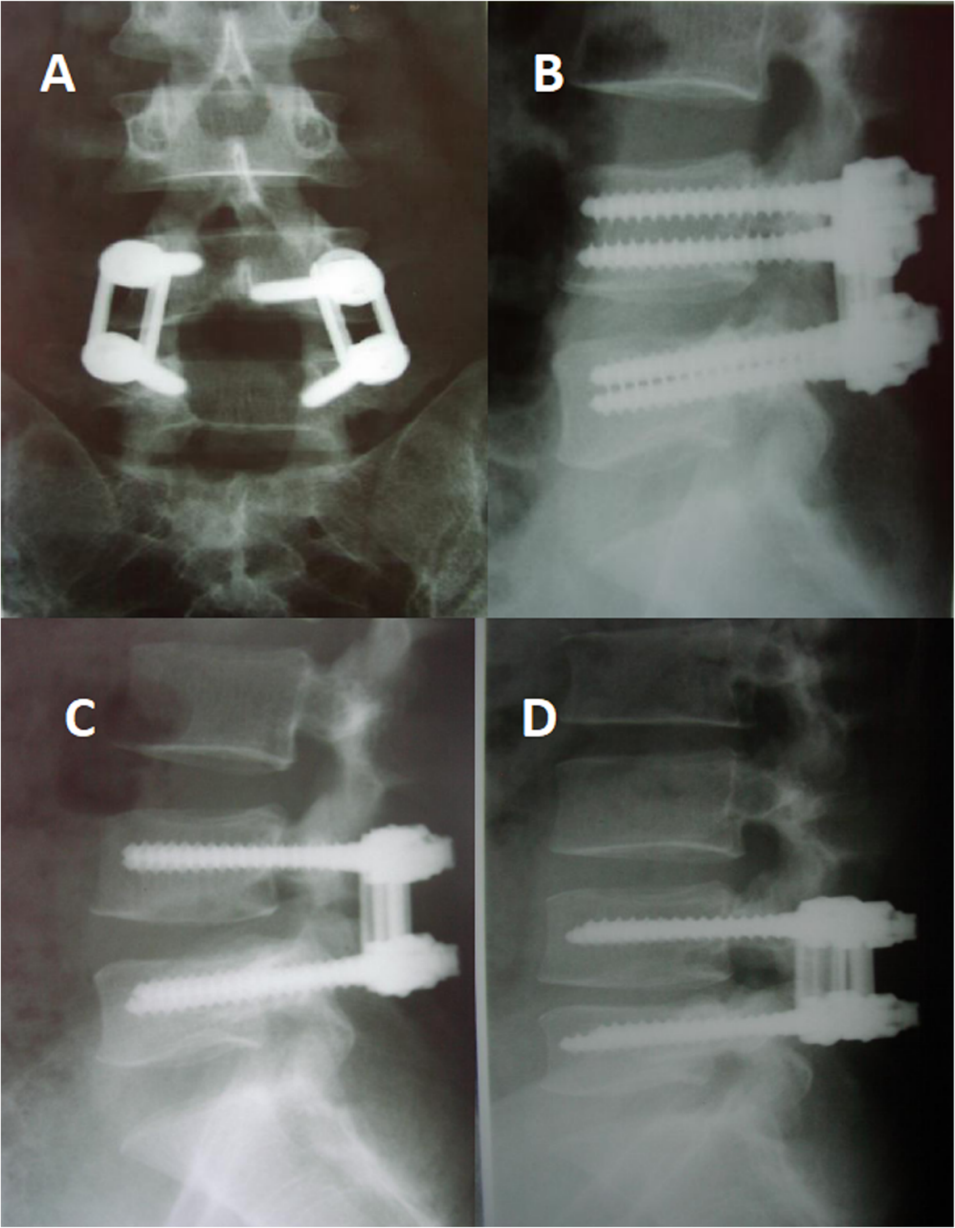
L4 DLS in a patient treated with L4 laminectomy and posterolateral fusion. Because of leg pain, she underwent revision surgery (**ab**). Postoperative X-ray showed screw displacement. **c** Postoperative X-ray showed that the screw was corrected and the pain improved. **d** L4-L5 disc height narrowing and posterolateral fusion were observed at the 48-month follow-up

**Fig. 2:**
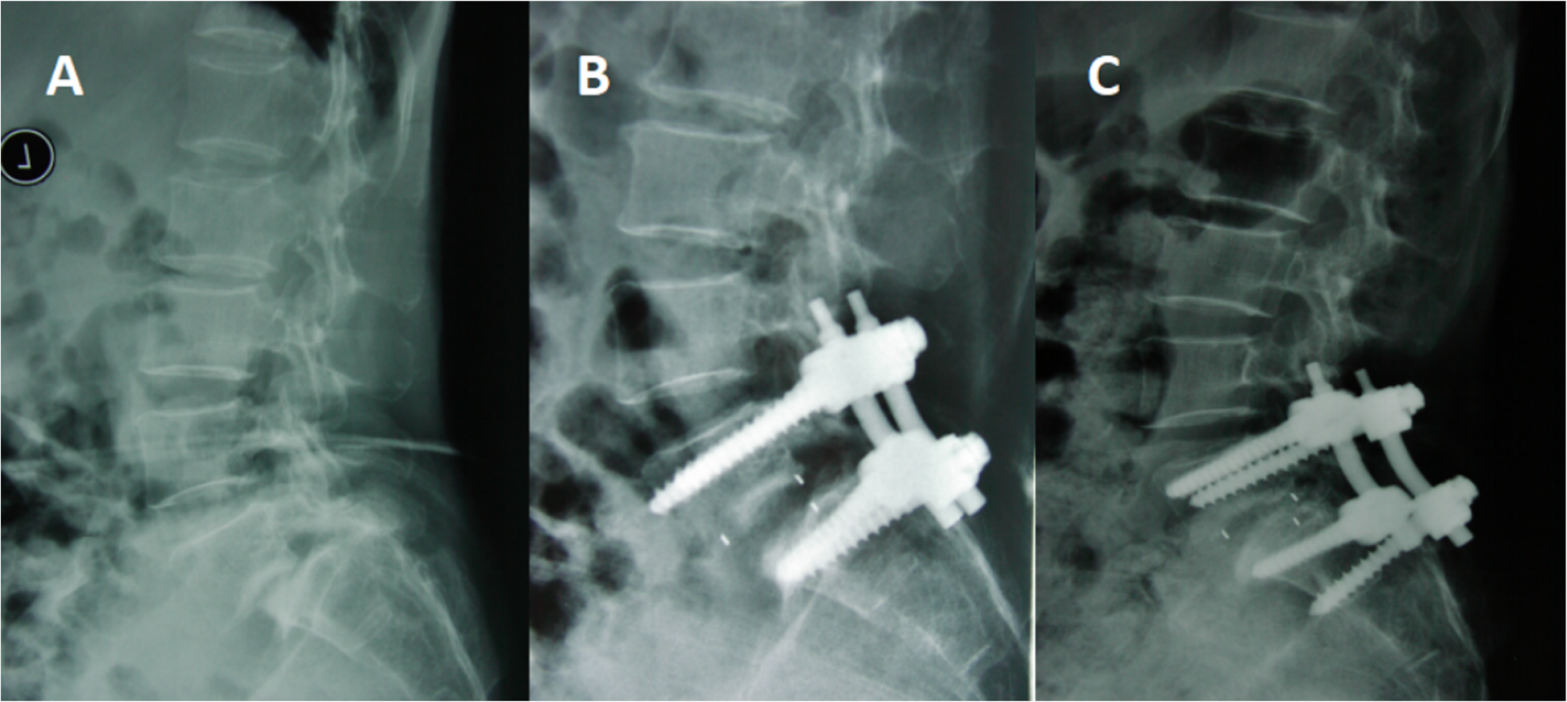
L5 DLS in a patient treated with L5 laminectomy and intervertebral interbody fusion. (**a, b**) Postoperative X-ray was normal. **c** The X-ray showed screw displacement and bone union at the 52-month follow-up

**Fig. 3:**
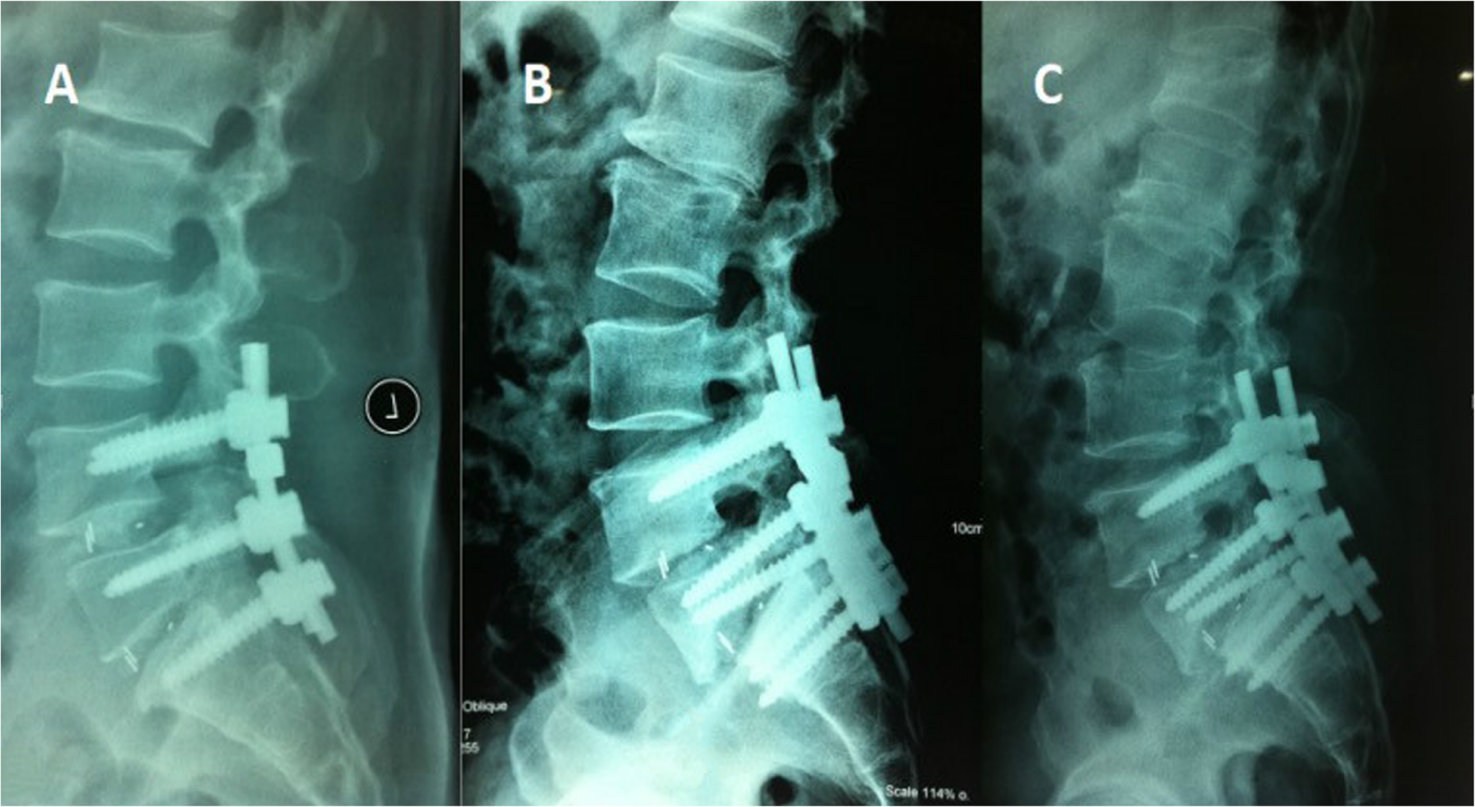
L4 and L5 DLS in a patient treated with L4/5 laminectomy and intervertebral interbody fusion (L4–5, L5-S1). **a** The postoperative X-ray was normal. **b** The X-ray showed bone union and screw displacement, and the patient refused revision surgery at the 12-month follow-up. **c** The X-ray showed that screw displacement and bone union worsened at the 2-year follow-up. The patient suffered from leg pain, and the patient underwent reoperation

**Fig. 4:**
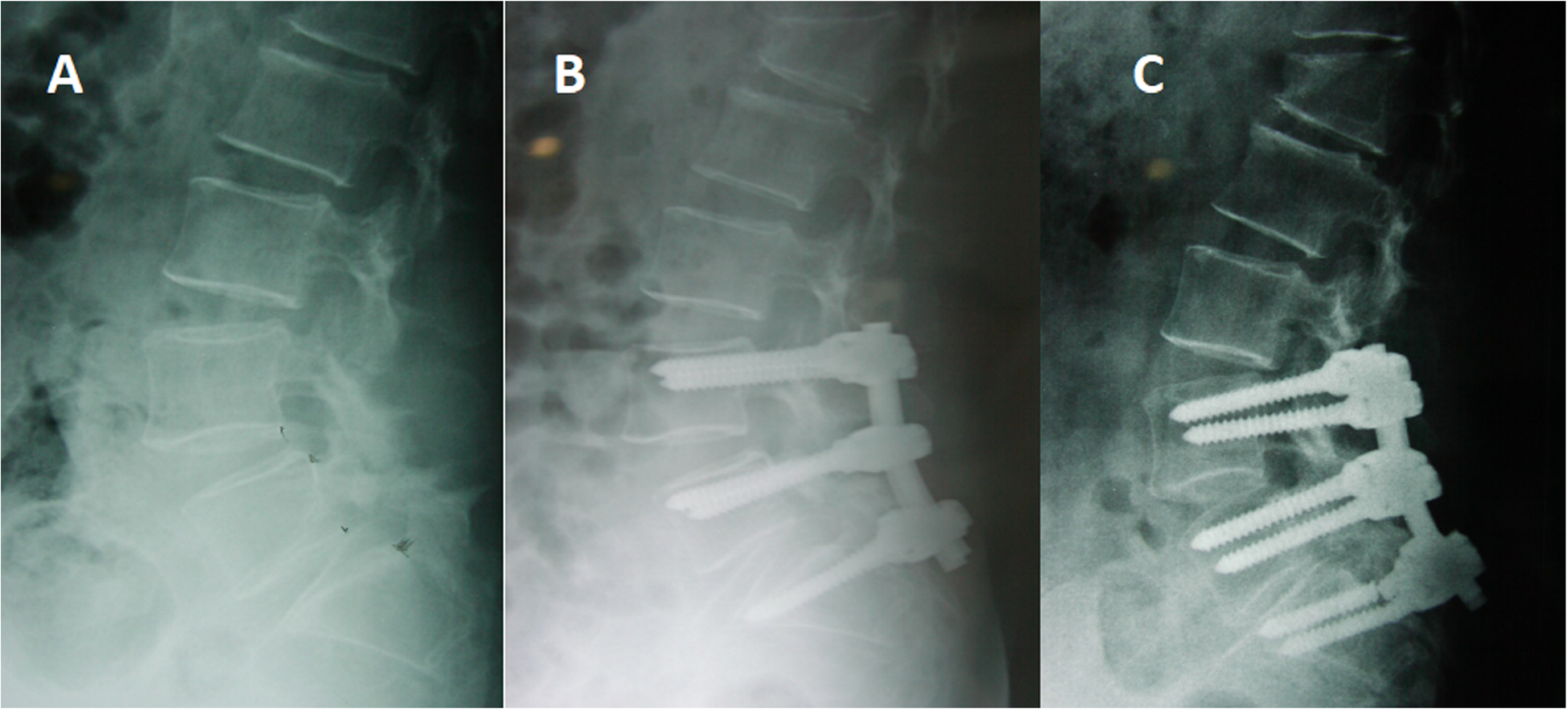
L4 DLS in a patient treated with L5 laminectomy and posterolateral fusion. (**a, b**) Postoperative X-ray was normal. **c** The X-ray showed screw displacement and fracture at the 36-month follow-up

A total of 33 patients underwent unplanned reoperations (3%). The patients were followed for an average of 4.20 ± 2.25 years (group A) and 4.32 ± 2.54 years (group B) without significant differences. Significant differences were found in the mean age, levels of involvement, hospital stay, surgery time, and blood loss between the groups (Table 1). The causes of unplanned operation were wound infection, screw misplacement (Fig. 1), neurological deficit, nonunion (Figs. 2 and 3), and screw fracture (Fig. 4), which were significant except for wound infection between the groups. Cases of screw misplacement, neurological deficit, nonunion and screw fracture in group A were more significant than those in group B.

**Table 1:** Comparison of the baseline data

	Posterolateral fusion	Intervertebral fusion	*P* value
Patients (n)	24 (24/650)	9 (9/450)	
Males/females	9/15	3/6	
Mean age (y)	72.80 ± 11.7	71.90 ± 10.7	0.583
Follow-up (y)	4.20 ± 2.25	4.32 ± 2.54	0.654
Involved levels			
L4–5	18	5	< 0.0001
L5-S1	6	4	< 0.0001
Hospital stay (days)	12.55 ± 3.35	15.43 ± 5.20	< 0.0001
Surgery time (minutes)	100.50 ± 30.50	150.50 ± 25.50	< 0.0001
Blood loss (ml)	150.65 ± 35.45	230.90 ± 101.50	< 0.0001
Wound infection	4	3	0.730
Screw misplacement	7	3	< 0.0001
Neurological deficit	5	1	< 0.0001
Nonunion	4	1	< 0.0001
Screw fracture	4	1	< 0.0001

Wound infections were found in patients after debridement surgery without implant removal, and these patients were treated with antibiotics sensitive to the bacteria. Pedicle screw displacement was detected on postoperative X-ray from patients who manifested leg pain or persistent sciatica after the primary surgery. All of the screws were corrected immediately; the leg pain improved after the reoperation and recovered to normal at the final follow-up.

### Risk factor analysis

Based on the previous documents, the potential factors were selected and assessed for analysing risk factors using the following data: age, sex, body mass index (BMI), and fusion surgical methods. Higher BMI, diabetes mellitus (DM), more bleeding and female sex predicted a higher occurrence of wound infection (Table 2). Different fusion methods and female sex predicted the development of nonunion and screw fracture.

**Table 2:** Risk analysis for wound infection

Valuables	OR	*P* value	95% confidence interval	
BMI (obesity)	4.42	0.01	1.5	14.06
Sex (female)	3.82	0.015	1.05	9.73
Diabetes mellitus	3.08	0.025	0.78	7.85
Age	3.65	0.02	0.96	1.45
Fusion method	4.02	0.010	2.01	16.21
More bleeding	3.01	0.03	0.65	7.21

*BMI* Body mass index

## Discussion

With the increase in the elderly population in China, an increasing number of elderly patients with degenerative lumbar disease (DLD) require surgical treatment. DLS is a type of spondylolisthesis with an intact neural arch. The treatment objectives of surgery include neural decompression, motion segment stabilization, intervertebral disc height reconstruction and sagittal balance restoration [1, 2, 9–12]. Spondylolytic segment stabilization relies on fusion through an anterior, a posterior or a combined approach [12, 13]. The PLF technique has gained reliability and popularity for DLS. Posterolateral fusion or intervertebral interbody fusion immediately and rapidly reconstructs a biomechanically stable spine, thereby increasing the chance of fusion [14–16]. However, in elderly patients, it is a challenge to perform spine surgery because of the increasing presence of medical diseases and surgical complications [16]. Furthermore, reoperation is an unplanned event for these patients, their families and surgeons, which results in additional perioperative complications, including death and potential medical risks or medical disputes. It is important for surgeons to identify risk factors in order to make better preoperative decisions and evaluate the surgical procedures to avoid unplanned reoperations.

Several previous studies reported reoperation following primary lumbar surgery for degenerative conditions and indicated that the revision rates were 14.0% in the 1997 to 2000 cohort and 12.4% in the 1990 to 1993 cohort [17]. Ghogawala demonstrated that the reoperation rate after only decompression for DLS at 1 year postoperatively was 15% [18], while Blumenthal reported a rate of 37.5% at a mean follow-up of 3.6 years [19]. The reoperation rate found in the present study was nearly the same as that in previous studies: the reoperation rate for only decompression at the 1-year follow-up was 10.8%, and the rate increased to 29.7% at 5 years postoperatively and to 33.4% at the final follow-up [20, 21]. Another report demonstrated that the cumulative reoperation rate was 6.1% at 1 year, 8.5% at 2 years, 15.2% at 3 years, 17.7% at 5 years, and 23.3% at the final follow-up [22]. In contrast to the studies mentioned above, in our study, the reoperation rate of DLS was 3.0%, which was lower than the rates found in previous studies. The reason for the lower reoperation rate in our study may be that we only reported reoperation during patients with one-level or two-level DLS.

Elderly patients have commonly been considered to be at a higher risk of postoperative complications from DLS than younger patients. An approximately10–30% complications rate has been documented in patient who undergo surgery for DLS [23–27]. In the study by Okuda, postoperative complications were found in 16% of elderly patients after PLIF with pedicle screw placement [28]. In our series, the major reasons for reoperation were wound infection, screw misplacement, neurological deficit, nonunion, and screw fracture, which were similar to previous reports. Therefore, a better understanding of the complications, the risk factors and unplanned reoperation rate are helpful for improve surgical outcomes.

In our study, higher BMI, DM, more bleeding and female sex predicted the occurrence of wound infection, which other studies have previously reported [20, 21, 23]. Deep postoperative wound infection will significantly impact the surgical outcomes of DLS and will have an important influence on the surgical plan, which requires revision surgery with implant removal if necessary. This complication also increases the cost of hospitalization and nursing. Additionally, spine surgeons should pay more attention to improving surgical training and may need morehelp from multiple disciplinary teams (MDTs). Another common reason for unplanned reoperation was screw displacement in the study. Complications related to screw displacement in spinal surgery are common and often result in spinal cord and nerve root injuries as well as dural lesions that require immediate revision surgery. Fortunately, all neurological defects in the patients after reoperation were alleviated and returned to normal at the final follow-up. Additional spine courses or navigation systems may help reduce the rate of screw displacement. Additionally, we found that patients who underwent posterolateral fusion procedures had a higher incidence of nonunion and a higher rate of unplanned reoperation. The results showed that a detailed preoperative plan and strict follow-up may work for patients with DLS when indicated for surgical treatment.

In our study, the goals of early treatments for DLS were the decompression and posterolateral fusion. Due to the limitations of the concept, surgical technology and instrumentation materials [23, 27], more complications occurred which was one of the limitations of our study. With the development of spine biomechanics and the progress of materials, the decompression and solid intervertebral fusion are the key role for DLS which are indicated for surgery. However, if the elderly patients with more medical diseases, the decompression or/and posterolateral fusion may be a better choice. However, the patients still face different surgical options.

## Conclusion

The unplanned reoperation rate in elderly patients who underwent surgery for DLS was 3%. Patients with higher BMI, DM, older age, posterolateral fusion, and female sex predicted a higher incidence of unplanned reoperations. Spine surgeons may need to pay more attention to their preoperative training and to improving the surgical techniques of DLS treatment, which could reduce the reoperation rate. However, we want to point out that there are several limitations in this study. First, the study did not consider the distribution of one-level or two-level DLS in each group, which was associated with bias. Second, the retrospective nature of the small-sample study may be associated with bias. Third, the surgical option for patients was associated with bias. In the future, prospective, randomized studies with long-term follow-up periods are needed.

## Data Availability

The datasets generated and/or analysed during the current study are not publicly available because the data are confidential patient data, but they are available from the corresponding author upon reasonable request.

## Abbreviations

PLF: Posterior lumbar fusion
DLS: Degenerative lumbar spondylolisthesis
BMI: Body mass index
DM: Diabetes mellitus
DLD: Degenerative lumbar disease
MDT: Multiple disciplinary team
